# Alzheimer's Disease and Non-Demented High Pathology Control Nonagenarians: Comparing and Contrasting the Biochemistry of Cognitively Successful Aging

**DOI:** 10.1371/journal.pone.0027291

**Published:** 2011-11-07

**Authors:** Chera L. Maarouf, Ian D. Daugs, Tyler A. Kokjohn, Douglas G. Walker, Jesse M. Hunter, Jane C. Kruchowsky, Randy Woltjer, Jeffrey Kaye, Eduardo M. Castaño, Marwan N. Sabbagh, Thomas G. Beach, Alex E. Roher

**Affiliations:** 1 The Longtine Center for Neurodegenerative Biochemistry, Banner Sun Health Research Institute, Sun City, Arizona, United States of America; 2 Department of Microbiology, Midwestern University, Glendale, Arizona, United States of America; 3 Laboratory of Neuroinflammation, Banner Sun Health Research Institute, Sun City, Arizona, United States of America; 4 Department of Pathology, Oregon Health & Science University, Portland, Oregon, United States of America; 5 Layton Aging and Alzheimer's Disease Center, Department of Neurology, Oregon Health and Science University, United States of America; 6 Fundacion Instituto Leloir, Buenos Aires, Argentina; 7 Cleo Roberts Center for Clinical Research, Banner Sun Health Research Institute, Sun City, Arizona, United States of America; 8 Civin Laboratory for Neuropathology, Banner Sun Health Research Institute, Sun City, Arizona, United States of America; National Institute on Aging Intramural Research Program, United States of America

## Abstract

The amyloid cascade hypothesis provides an economical mechanistic explanation for Alzheimer's disease (AD) dementia and correlated neuropathology. However, some nonagenarian individuals (high pathology controls, HPC) remain cognitively intact while enduring high amyloid plaque loads for decades. If amyloid accumulation is the prime instigator of neurotoxicity and dementia, specific protective mechanisms must enable these HPC to evade cognitive decline. We evaluated the neuropathological and biochemical differences existing between non-demented (ND)-HPC and an age-matched cohort with AD dementia. The ND-HPC selected for our study were clinically assessed as ND and possessed high amyloid plaque burdens. ELISA and Western blot analyses were used to quantify a group of proteins related to APP/Aβ/tau metabolism and other neurotrophic and inflammation-related molecules that have been found to be altered in neurodegenerative disorders and are pivotal to brain homeostasis and mental health. The molecules assumed to be critical in AD dementia, such as soluble or insoluble Aβ40, Aβ42 and tau were quantified by ELISA. Interestingly, only Aβ42 demonstrated a significant increase in ND-HPC when compared to the AD group. The vascular amyloid load which was not used in the selection of cases, was on the average almost 2-fold greater in AD than the ND-HPC, suggesting that a higher degree of microvascular dysfunction and perfusion compromise was present in the demented cohort. Neurofibrillary tangles were less frequent in the frontal cortices of ND-HPC. Biochemical findings included elevated vascular endothelial growth factor, apolipoprotein E and the neuroprotective factor S100B in ND-HPC, while anti-angiogenic pigment epithelium derived factor levels were lower. The lack of clear Aβ-related pathological/biochemical demarcation between AD and ND-HPC suggests that in addition to amyloid plaques other factors, such as neurofibrillary tangle density and vascular integrity, must play important roles in cognitive failure.

## Introduction

The incidence of Alzheimer's disease (AD) is increasing worldwide and imposing enormous economic burdens [1]. Alzheimer's disease is the most common form of dementia, presently accounting for 5.5 million cases in the USA, a number projected to double by the end of this decade. Unprecedented advances in the biomedical field, hygiene and nutrition have increased the average life expectancy with an impressive exponential growth in the numbers of those surviving beyond 90 years, defined as the “oldest-old” [2]–[5]. The general health of aging populations is an urgent issue in terms of the overwhelming and mounting emotional burdens and expenses that the elderly generate. Awareness and intervention are promptly needed given the fact that AD dementia is reaching alarming proportions. Worldwide, there will be 1.3 billion people over the age of 65 years and the numbers of those over age 80 is predicted to increase by 233% by 2040 [6].

Alzheimer's disease is defined by the profuse deposition of amyloid-beta (Aβ) peptides in amyloid plaques and walls of cerebral vessels as well as by the accumulation of intracellular neurofibrillary tangles (NFT). These lesions are accompanied by synaptic depletion, neuronal demise, gliosis, demyelination and severe brain atrophy. The “amyloid cascade hypothesis” suggests that the production and profuse deposition of insoluble fibrillar amyloid and of increased soluble oligomeric forms of Aβ initiates a series of events culminating in neuronal damage, cognitive impairment and ultimately dementia [7], [8]. This hypothesis is supported by studies demonstrating higher probability of AD and an increasing number of amyloid plaques and NFT with increasing age [2], [9], [10]. The discovery of familial AD mutations in the amyloid precursor protein (APP) coding region, and APP processing genes presenilin-1 (PS-1) and presenilin-2 (PS-2) as well as subsequent work in transgenic mouse models carrying human APP and PS mutations also lent strong support to the amyloid cascade hypothesis [11]. Based on these observations, a large number of therapeutic interventions have been designed to prevent the generation of Aβ, reduce its deposition or remove already existing amyloid plaques.

While the amyloid cascade hypothesis is the prevailing mechanism to explain the pathogenesis of sporadic AD, amyloid plaque density has not been shown to robustly correlate with either AD diagnosis or as a measure of disease severity or progression [10], [12]. Levels of amyloid/Aβ determined by ^11^C-Pittsburgh compound B (PIB)-PET imaging, or altered plasma or CSF Aβ values have not yet been proven to adequately predict AD or cognitive decline, without being combined with other biomarkers of dementia such as tau [13]–[15]. As many as 30% of elderly individuals with no cognitive impairment have positive PIB/amyloid imaging signals [16], [17]. Furthermore, a significant proportion of elderly individuals exhibit sufficient plaque densities warranting a neuropathology-based classification as probable AD, yet were normal by cognitive assessments [10]. Some oldest-old individuals are able to remain cognitively intact and endure high amyloid plaque loads for years or even decades [17]. These observations cast doubts on the amyloid cascade hypothesis as the sole determinant of dementia and demand an explanation as to why some elderly individuals harbor such high levels of amyloid without cognitive impairment. If amyloid accumulation produces neurotoxicity and dementia, protective mechanisms must be in place to enable these individuals to evade cognitive decline. If, on the other hand, the amyloid cascade mechanism of dementia production is incorrect, why are amyloid plaques produced and what purpose do they serve?

In an attempt to resolve these conundrums, we are initiating a series of systematic studies to determine at the molecular level the differences and similarities between AD and non-demented oldest-old meeting the neuropathological criteria for AD. In the first of these studies, we assessed the neuropathological differences between these two groups. In addition, we quantified by ELISA and Western blots a group of proteins related to APP/Aβ/tau metabolism and other neurotrophic and inflammatory-related molecules that have been found to be altered in neurodegenerative disorders and that are pivotal to brain homeostasis and mental health.

## Materials and Methods

### Human subjects

Brain specimens were obtained from the Banner Sun Health Research Institute (BSHRI) Brain and Body Donation Program [18]. All cases were selected for advanced age (90 years and older) and were neuropathologically classified as having “moderate” or “frequent” CERAD neuritic plaque scores. In addition, they were free of other neurodegenerative disorders such as vascular dementia, Parkinson's disease, dementia with Lewy bodies, frontotemporal dementia, hippocampal sclerosis, progressive supranuclear palsy, dementia lacking distinctive histology, multiple system atrophy, motor neuron disease with dementia and corticobasal degeneration. Included in the study was a cohort of 8 individuals (cases 1–8) with a mean age of 92.8 years (range: 90–100 years) that were clinically assessed as non-demented (ND) as shown in **Table 1**. On neuropathological examination these cases contained sufficient AD amyloid plaque and neurofibrillary tangle density to meet at least NIA-Reagan “intermediate” neuropathological criteria for AD, and hence were classified as non-demented high pathology controls (ND-HPC). A second cohort of 6 demented individuals (cases 10–15), with a mean age of 94.2 years (range: 90–96 years) were confirmed by neuropathological examination as having at least NIA-Reagan “intermediate” AD and were free of other neuropathological diagnoses (**Table 1**; see below for additional information). The NFT score and Braak stage as well as the scores for cerebral amyloid angiopathy (CAA), white matter rarefaction (WMR) and apolipoprotein E (ApoE) genotype were not considered in the selection of these cases. The age, gender distribution, postmortem interval (PMI), brain weight, last Mini-Mental State Examination (MMSE) score, ApoE genotype, total plaque score, total tangle score, Braak stage, total WMR score and total CAA score of each individual in the ND-HPC and AD groups are presented in **Table 1**.

**Table 1:** Oldest-old BSHRI Study Subject Data.

ND-HPC	Expired age (y)	Gender	PMI (h)	Brain Weight (g)	Last MMSE score	ApoE GT	Total plaque score	Total tangle score	Braak stage	Total WMR score	Total CAA score
1	91	M	3.0	1050	-	3/4	10.75	5	III	0	0
2	100	M	2.5	1160	29	3/3	14	8	IV	1	8
3	90	F	4.3	975	28	3/3	10.5	5	III	1	0
4	94	M	3.5	1100	27	3/3	15	10.5	IV	10	9
5	90	F	2.5	966	25	3/3	13.5	8	IV	2	1
6	92	M	3.2	1300	27	2/4	14	12	V	1	8
7	91	M	4.3	1150	29	2/3	14.5	8.5	IV	1	1
8	94	M	2.5	1050	29	3/3	15	12	IV	2	1
**Mean**	**92.8**		**3.2**	**1094**	**27.7**		**13.4**	**8.6**		**2.3**	**3.5**

AD	Expired age (y)	Gender	PMI (h)	Brain Weight (g)	Last MMSE score	ApoE GT	Total plaque score	Total tangle score	Braak score	Total WMR score	Total CAA score
10	95	F	3.2	1040	16	3/4	12.2	10	VI	0	8
11	90	M	14.0	1300	19	3/3	11	6.25	IV	7	2
12	96	F	3.0	1000	18	3/3	13.75	8	IV	1	6
13	96	F	3.0	900	5	2/3	10	15	VI	12	12
14	96	F	3.3	960	13	3/4	11.5	15	VI	10	9
15	92	F	2.8	900	13	4/4	14.5	15	VI	3	2
**Mean**	**94.2**		**4.9**	**1017**	**14.0**		**12.2**	**11.5**		**5.5**	**6.5**

ND-HPC  =  non-demented high pathology controls; AD  =  Alzheimer's disease; y  =  years; M  =  male; F  =  female; PMI  =  postmortem interval; h  =  hours; g  =  grams; MMSE  =  mini-mental state examination; ApoE  =  apolipoprotein E; GT  =  genotype; WMR  =  white matter rarefaction; CAA  =  cerebral amyloid angiopathy.

### Neuropathological evaluation

Brain sections (40 µm thickness) were stained with Campbell-Switzer, Thioflavine-S, Gallyas and hematoxylin and eosin (H&E) to visualize amyloid deposits and NFT and the grade of WMR (leukoaraiosis). The clinicopathological diagnosis of AD was established by the presence of dementia and an NIA-Reagan rating of at least “intermediate” in terms of neuritic plaque density and Braak NFT stage [19]. Total plaque score for each brain was obtained by estimating the density of all plaque types including compact, neuritic, classical and diffuse revealed by Thioflavine-S and Campbell Switzer silver stains. Plaque densities were evaluated using the CERAD templates [20], [21] as none, sparse, moderate and frequent and reported numerically as 0, 1, 2 and 3, respectively. Five regions were appraised: frontal, temporal, parietal, hippocampal and entorhinal, to render a maximum score of 15. The total NFT score was assessed in the same fashion as described for the total plaque score, again using the published CERAD templates for this purpose. The Braak stage (I-VI) was estimated by the method described by Braak and Braak [22]. White matter rarefaction was evaluated in the frontal, temporal, parietal and occipital lobes on one quarter of hemisphere sections stained by H&E. The scores were none, mild (less than 25% affected), moderate (25–50% affected) and severe (greater than 50% affected) and were converted into numeric scores of 0, 1, 2, 3, yielding a maximum possible score of 12 [18]. The CAA score was ranked in a similar fashion as none, mild, moderate and severe (0, 1, 2 and 3) estimated in the cortical areas of the frontal, temporal, parietal and occipital lobes using Thioflavine-S staining. ApoE genotypes were obtained using the technique of Hixson and Vernier [23] on DNA isolated from cerebellar samples.

### Aβ, tau and α-synuclein ELISA quantification

All steps were performed at 4°C. Gray matter and white matter were dissected from frozen frontal lobe tissue (100 mg) and homogenized in 6 volumes (600 µl) of 20 mM Tris-HCl, 5 mM EDTA, pH 7.8, protease inhibitor cocktail (PIC, Roche Diagnostics, Mannheim, Germany) with a Teflon tissue grinder. The homogenate was centrifuged in a TLA 120.2 rotor (Beckman) for 20 min at 435,000 × *g*. The Tris-HCl-soluble supernatant was collected and total protein measured with the Micro BCA protein assay kit from Pierce (Rockford, IL). The remaining pellet was dissolved in 600 µl of 90% glass distilled formic acid (GDFA) with an electric grinder (Omni TH, Kennesaw, GA) and incubated for 1 h. The GDFA homogenates were then centrifuged at 435,000 × *g* in a TLA 120.2 rotor for 20 min. The supernatant was collected and dialyzed 3 times, 30 min each against deionized water then twice for 1 h against 0.1 M ammonium bicarbonate and lyophilized. The lyophilized material was reconstituted in 500 µl 5 M guanidine hydrochloride (GHCl), 50 mM Tris-HCl, pH 8.0, PIC (Roche), shaken for 3 h, centrifuged at 435,000 × *g* in a TLA 120.2 rotor for 20 min, the supernatant collected and total protein determined with Pierce's Micro BCA protein assay kit. Aβ40, Aβ42, tau and α-synuclein were quantified with ELISA kits from Invitrogen according the manufacturers' instructions.

### Tumor necrosis factor-α (TNF-α) ELISA quantification

All steps were performed at 4°C and have been described in detail [24]. Briefly, frozen frontal lobe gray matter (100 mg) was homogenized in 1 ml 20 mM HEPES, 1.5 mM EDTA, pH 7.4, PIC (Roche), centrifuged at 3000 × *g* and the supernatant centrifuged again at 40,000 × *g* (TLA 120.2 rotor). The resulting supernatant was submitted to Pierce's Micro BCA protein assay for total protein determination. A kit from PromoKine (Heidelberg, Germany) was used to quantify human TNF-α levels following the manufacturer's instructions.

### CD200 ELISA

Frozen frontal lobe gray matter samples were extracted in 5 volumes of RIPA buffer (25 mM Tris-HCl (pH 7.6), 150 mM NaCl, 1% sodium deoxycholate, 0.1% SDS) containing Halt proteinase and phosphatase inhibitor mixture (Thermo Scientific, Pierce) for 30 min. The supernatant resulting from centrifugation (18,000 × *g*/30 min) was assayed for total protein concentration. The CD200 ELISA used two monoclonal antibodies to CD200 (R&D Systems, Minneapolis, MN). The capture antibody was used at 1 µg/ml and the biotinylated detection antibody was used at 50 ng/ml. Samples were added to plates at 1.5 µg/well (100 µl) diluted in phosphate buffered saline (PBS) containing 0.05% Tween-20 (PBST) and 1% BSA (sample diluent). Plates were blocked with sample diluents, the samples and standards incubated on plates for 2 h at room temperature and the plates washed using an automated plate washer. The detection antibody was added and incubated for 2 h. After further washing, bound immune complexes were detected by incubation in 1∶200 dilution of Streptavidin-HRP (R&D Systems). Plates were developed with tetramethylbenzidine (TMB) ELISA substrate for 20 min and after the reaction was terminated with 1 M sulfuric acid and the absorbance of each well read by spectrophotometry at 450 nm. A standard curve was constructed using values of diluted CD200 recombinant purified protein (R&D Systems). Amounts of CD200 protein in each sample were calculated from the standard curve.

### Synaptophysin ELISA quantification

Synaptophysin protein concentrations were measured in each sample using an ELISA. Gray matter samples (frozen frontal lobe) were extracted in 10 volumes of RIPA buffer (Thermo Scientific), centrifuged at 18,000 × *g* for 30 min and the supernatants adjusted to 15 mg/ml protein concentration. Plates were coated with 1∶1000 dilution of monoclonal antibody to synaptophysin (SP-17 – Covance Research Products, Princeton, NJ) as capture antibody. Samples were applied to ELISA plates along with dilutions of recombinant synaptophysin standard (AbNova, Taipei, Taiwan). Samples and standard were incubated overnight at 4°C. Plates were washed and bound synaptophysin quantified by sequential incubations with detection antibody (Millipore, Temecula, CA), horseradish peroxidase (HRP) labeled anti-rabbit immunoglobulin and TMB ELISA substrate. Reactions were terminated after 30 min with 1 M sulfuric acid and absorbances measured at 450 nm. The concentration of synaptophysin in each sample was calculated by comparison with the standard curve.

### GFAP ELISA

Sample preparation was the same as for the CD200 ELISA samples. The glial fibrillary acidic protein (GFAP) ELISA used a pool of monoclonal antibodies to GFAP as capture antibodies (0.25 µg/ml; BD Biosciences) and a rabbit polyclonal to GFAP as detection antibody (1∶10,000 dilution - DAKO). Samples were added to plates at 15 ng/well (100 µl), diluted in PBST and 1% skimmed milk (sample diluent). Plates were blocked with sample diluent and the specimens and standards were incubated on plates for 2 h at room temperature and washed using an automated washer. The detection antibody was added and incubated for 2 h. After further washing, bound immune complexes were detected by incubation in 1∶20,000 dilution of HRP labeled anti-rabbit immunoglobulin (Pierce). Plates were developed with TMB ELISA substrate and after the reaction was terminated with 1 M sulfuric acid, the absorbance of each well read by spectrophotometry at 450 nm. A standard curve was constructed using values of diluted GFAP purified protein (EMD Merck). Amounts of GFAP protein in each sample were calculated from the standard curve.

### ApoE ELISA quantification

A human ApoE ELISA was performed as previously described [25]. ELISA plates were coated with goat anti-human ApoE (1∶2000 dilution in PBS, Millipore), washed 1X with PBST, blocked (PBST, 1% BSA, 50 mM glycine, PIC (Roche)), then washed 3X with PBST. Tris-soluble and GHCl-soluble gray matter samples were prepared as described for Aβ, tau and α-synuclein ELISA quantification (see above) and were diluted 1∶2500 in blocking buffer. RIPA and 5% SDS soluble samples from BSHRI were prepared as described in the Western blot analysis section (see below). RIPA and 5% SDS soluble samples were diluted 1∶5000 and 1∶10,000 respectively in blocking buffer. Samples (100 µl/well) were then added to the ELISA plates and incubated overnight at 4°C. Plates were washed 4X with PBST and incubated for 2 h at 4°C with 100 µl/well of detection antibody (biotinylated goat anti-ApoE, 1∶2000 dilution in blocking buffer, Meridian Life Sciences). The plates were washed 4X with PBST, incubated for 1 h at 4°C with 100 µl of Streptavidin–HRP reporter (1∶20000 dilution in PBST, Invitrogen), washed 4X with PBST, incubated with 100 µl of TMB substrate for 8 min, stopped with 100 µl of 1 N sulfuric acid and read at 450 nm.

### Western blot analysis

All steps were performed at 4°C (except for 5% SDS homogenates) and all materials were from Invitrogen and chemicals from Sigma unless otherwise noted. One-hundred mg of frozen gray matter from the frontal lobe was homogenized in 1 ml of RIPA buffer (Sigma) containing PIC and PhosSTOP (phosphatase inhibitor cocktail, Roche) using an Omni TH electric grinder. The samples were centrifuged at 14,000 × *g* for 20 min in a Beckman 22R centrifuge, the supernatant was recovered and total protein determined with a Micro BCA protein assay (Pierce). Alternatively, 100 mg of gray matter was homogenized in 1 ml of 5% SDS, 5 mM EDTA, 20 mM Tris-HCl, pH 7.8 with the Omni TH electric grinder. The samples were centrifuged as described above and Pierce's Micro BCA protein assay kit used the measure total protein. A total of 10 µg, 20 µg or 40 µg of total protein was brought up to 15 µl with NuPage 2XLDS sample buffer, 50 mM dithiothreitol (Sigma) then incubated for 10 min at 80°C. The proteins were separated on 15 well 4–12% Bis-Tris gels with NuPage 1XMES run buffer supplemented with NuPage antioxidant. The Kaleidoscope prestained marker (Bio-Rad, Hercules, CA) was loaded onto each gel as a molecular weight standard. The proteins were then transferred onto nitrocellulose membranes (0.45 µm pore) with NuPage transfer buffer and 20% methanol (Pharmco-Aaper). The membranes were blocked in 5% Quick-Blocker (G-Biosciences) in PBS (EMD Chemicals, Gibbstown, NJ), 0.5% Tween 20. Primary and secondary antibodies were diluted in the same blocking buffer. **Table 2** lists the antibodies applied for these experiments. SuperSignal WestPico Chemiluminescent (Pierce) substrate, CL-Xpose film (Pierce) and Kodak GBX developer and fixer were used to detect the proteins. To control for any inadvertent differences in total protein loading, antibodies were stripped from the membranes with Restore™ Western Blot Stripping Buffer (Pierce). After washing, the membranes were re-blocked and re-probed with anti-mouse or anti-rabbit actin antibody **(** **Table 2** **)**. A GS-800 calibrated densitometer (Bio-Rad) and Quantity One software (Bio-Rad) were used to scan and analyze the films. The trace quantity feature in Quantity One was used to appraise the density of each band and refers to the measured area under each band's intensity profile curve. The units are in optical density (OD) x mm.

**Table 2:** Primary and Secondary Antibodies Used in Western Blots.

Primary Antibody (WB)	Antigen specificity or immunogen	Secondary antibody	Company/Catalog #
22C11	APP aa 66–81	M	Millipore/MAB348
CT9APP	Last 9 aa of APP	R	Millipore/AB5352
4G8	Aβ aa 17–24	M	Covance/SIG-39220
6E10	Aβ aa 1–16	M	Covance/SIG-39320
A11	Sequence independent oligomers	R	Invitrogen/AHB0052
BACE1	BACE1 aa 485–501	R	Abcam/ab2077
IDE (BC2)	Rat IDE aa 97–273	R	Provided by Dr. E. Castaño
Neprilysin	Rat neprilysin	R	Millipore/AB5458
Notch-1	NICD N-terminal 14 aa	R	Millipore/AB5709
Tau (HT7)	Tau aa 159–163	M	Pierce/MN1000
PHF-tau AT8	pTau Ser202	M	Pierce/MN1020
PHF-tau AT180	pTau Thr231	M	Pierce/MN1040
α-synuclein	Rat synuclein-1 aa 15–123	M	BD Transduction Laboratories/610786
ApoE	Recombinant ApoE	G	Millipore/AB947
ApoJ	Recombinant ApoJ	G	Millipore/AB825
VEGF165	Recombinant human VEFG165	R	Millipore/07-1419
PEDF	Human PEDF	R	BioProducts MD/AB-PEDF1
BDNF/proBDNF	Internal region of BDNF	R	Santa Cruz/sc-546
TDP-43	aa residues surrounding Ala260 of human TDP-43	R	Cell Signaling Technology/3449
Synaptophysin	Rat retina synaptophysin	M	Millipore/MAB368
S100B	C-terminal synthetic peptide of human S100B	R	Abnova/PAB13687
Actin Ab-5	Clone C4	M	BD Transduction Laboratories/A65020
Actin	N-terminus of human α-actin	R	Abcam/Ab37063

APP, amyloid-β precursor protein; aa, amino acid; BACE, β-site APP cleaving enzyme; IDE, insulin degrading enzyme; NICD, Notch-1 intracellular domain; PHF, paired helical filament; ptau, phosphorylated tau; VEGF, vascular endothelial growth factor; PEDF, pigment epithelium derived factor; BDNF, brain derived neurotrophic factor; TDP-43, TAR DNA-binding protein 43; ApoE, apolipoprotein E; ApoJ, apolipoprotein J; M, HRP conjugated AffiniPure goat-anti mouse IgG (catalog # 111-035-144, Jackson Laboratory); R, HRP conjugated AffiniPure goat-anti rabbit IgG, (catalog # 111-035-146 Jackson Laboratory); G, HRP conjugated AffiniPure bovine-anti goat IgG (catalog #805-035-180).

## Results and Discussion

A large number of studies have compared the structural and biochemical differences that exist between AD and ND control individuals with minimal AD pathology. Our study represents an initial exploration into the molecular and neuropathological conditions that prevail in the oldest-old AD and ND-HPC, which are of paramount importance in understanding the etiology of AD. The study involved uncomplicated AD cases with overt dementia and high amyloid plaque burdens. In a similar fashion we selected as a control population, cases with high MMSE scores and a high amyloid plaque load (ND-HPC). In both groups we allowed the rest of the neuropathological AD lesions and genetics to behave as unknown variables. In this investigation we are trying to address some basic questions such as 1) Is AD dementia caused by neurotoxic amyloid plaques and NFT? 2) Are plaques and NFT the result of the aging process in the oldest-old? 3) Do the ND-HPC have some specific increase or decrease molecular expression that confers protection from dementia?

### I. Human subjects and Neuropathology analyses

**Figure 1:**
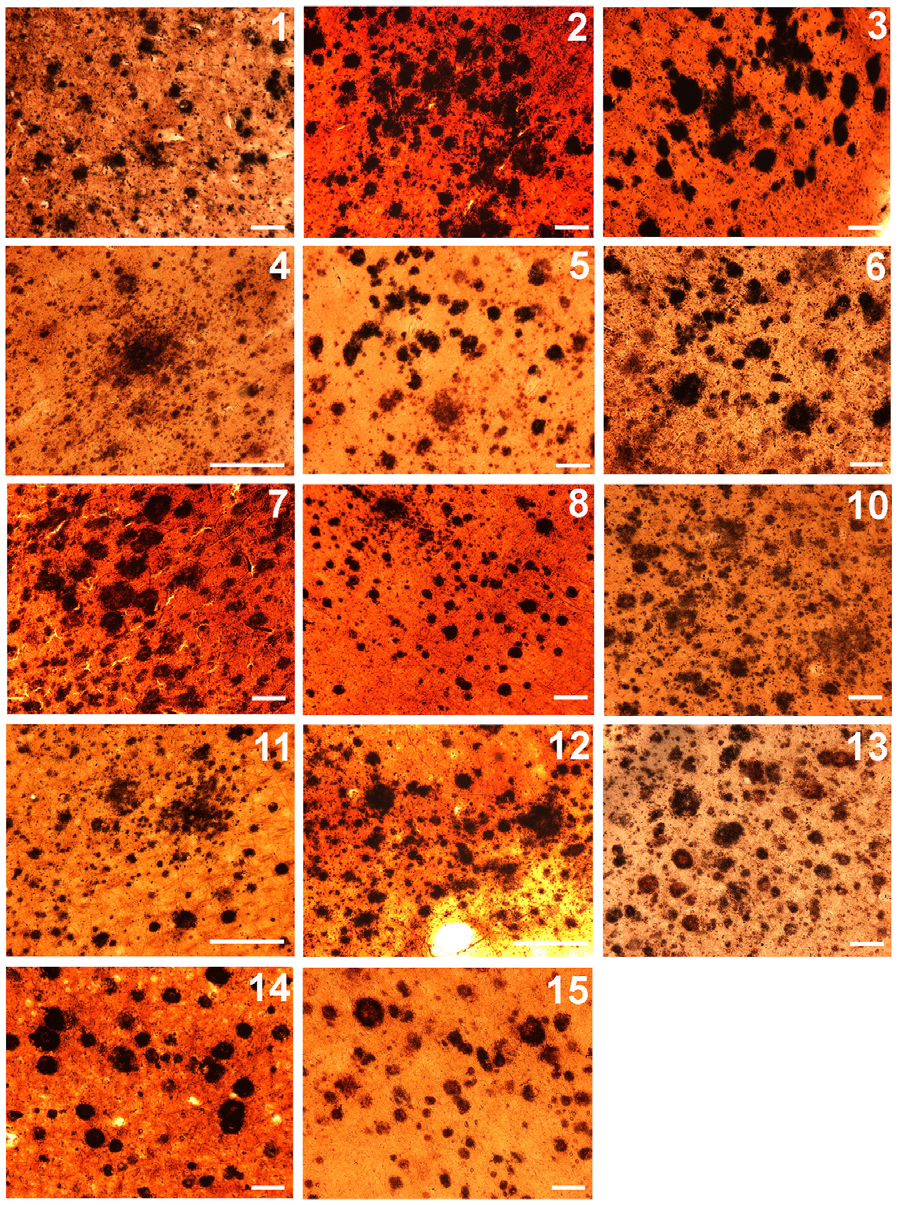
Amyloid plaques stained by the Campbell-Switzer technique. The histological fields are representative of the amyloid plaque burden shown in 40 µm coronal sections of the frontal lobe. The numbers on the top right corners correspond to the case identification numbers given in **Table 1**. Scale bars  = 100 µm.

The average age of the oldest-old groups was 93 years for the ND-HPC (n = 8) and 94 years for the AD individuals (n = 6). In reference to gender distribution there was a preponderance of males in the ND-HPC (6 males and 2 females) with the opposite in the AD group (5 females and 1 male) **(** **Table 1** **)**. The postmortem interval was 3.2 h and 4.9 h for the ND-HPC and AD groups, respectively. A total plaque numeric score was calculated which accounted for the occurrence of all types of plaques (compact, neuritic, classical and diffuse) and evaluated separately for each of the four cerebral lobes. In addition, an overall neuritic plaque density was calculated according to the CERAD guidelines as the highest density achieved in any of the four lobes **(** **Table 1** **)**. As the oldest-old ND-HPC and AD subjects were selected for abundant amyloid plaque loads, their total plaque scores were similar 13.4 and 12.2 for ND-HPC and AD cases, respectively. The relative plaque densities between these two cohorts are illustrated in **Figure 1**. From a neuropathological point of view, the ND-HPC and AD cases were purposefully selected for abundant amyloid plaques. However, the load of vascular amyloid (total CAA score), which was not used in the selection of cases, was on the average almost 2-fold more abundant in AD than in the ND-HPC **(** **Table 1** **)**, suggesting that a higher degree of microvascular dysfunction and perfusion compromise was present in the demented cohort. The ApoE allelic distribution in the ND-HPC and AD groups was: ApoE2 = 0.125, ApoE3 = 0.75 and ApoE4 = 0.125, and ApoE2 = 0.08, ApoE3 = 0.58 and ApoE4 = 0.33, respectively **(** **Table 1** **)**.

**Figure 2:**
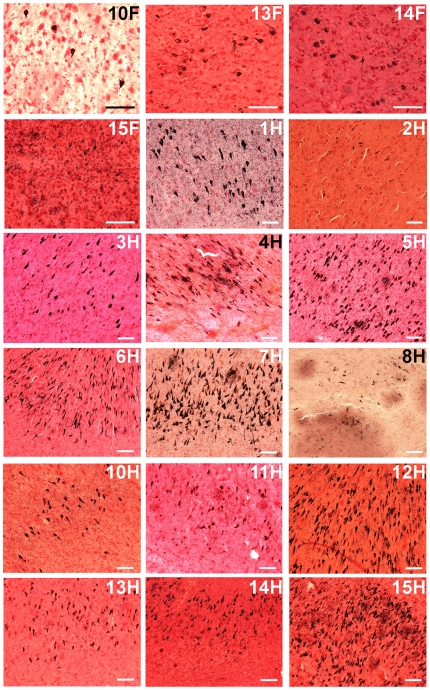
Neurofibrillary tangles stained by the Gallyas technique. The histological fields are representative of the neurofibrillary tangle abundance shown in 40 µm coronal sections of the frontal lobe and hippocampus. The numbers on the top right corners correspond to the case identification numbers given in **Table 1** and the letters F and H represent frontal cortex and hippocampus, respectively. Scale bars  = 100 µm.

In reference to the NFT distribution, the AD group demonstrated a more advanced Braak stage than the ND-HPC **(** **Table 1** **)**. Intriguingly, the ND-HPC showed no NFT in the frontal lobe (data not shown), while in the AD group 4 out of 6 cases harbored NFT in this region (**Figure 2** **, 10F, 13**–**15F)**. Appraisal of NFT in the hippocampus revealed moderate to abundant levels in both the ND-HPC and AD groups, with the exception of AD case # 11 and ND-HPC cases # 2 and # 8 **(** **Figure 2** **,** **1** **-8H, 10**–**15H)** in which the NFT were scarce. Disparities were likewise evident for total cerebral NFT scores, which were about 25% higher in the AD subjects than in the ND-HPC **(** **Table 1** **)**. It is possible that in the oldest-old ND-HPC the absence of NFT in the frontal cortex allows for a better performance in terms of executive function, strategic planning and cognitive tasks. In addition, a decreased tangle density suggests fewer injured neurons, correspondingly less brain atrophy and better brain function in the oldest-old ND-HPC [5], [26], [27]. Recent observations in the transgenic mice Tg4510 strain, carrying the frontotemporal dementia tau P301L mutation, suggest that NFT are a marker rather than the direct cause of neuronal dysfunction and death. In this model, tangle deposition is apparently preceded by caspase activation which has been associated with acute apoptotic death [28]. Our own electron microscopic observations on AD brain biopsies suggest NFT are derived from collapsing mitochondria and other intraneuronal pathological organelles, supporting the contention that NFT are the result of damaged cytomembranes [29]. Chemical analysis of the protease resistant core of paired helical filaments revealed associated glycolipids [30], [31] that could originate from membrane walls.

A more pronounced difference was observed in the WMR category where the mean values for the ND-HPC and AD were 2.3 and 5.5 (out of a maximum total of 12), respectively **(** **Table 1** **)**. Cerebral WMR, also known in the imaging literature as “leukoaraiosis”, “white matter lucencies” on CT, “white matter hyperintensities” on MRI or “small vessel ischemic disease” probably has at least two etiologies. It may represent secondary white matter degeneration occurring after primary cortical gray matter disease or “incomplete infarction” of white matter due to circulatory insufficiency. The ischemic mechanism has received the most attention, particularly in the imaging literature, where it has been hypothesized that WMR results from brain hypoperfusion. This is thought to particularly affect the periventricular areas that are supplied by the terminal branches of the deep perforating arteries. In a recent study, cardiac output (ml of ejected blood per min) and the severity of white matter hyperintensities were found to be inversely correlated, after adjustment for confounding factors [32], suggesting that cardiovascular system decline is associated with loss of axons and myelin. Furthermore, in the elderly, decreased cardiac index (cardiac output/body surface area) was correlated with reductions in brain volume which may be associated with decreased brain perfusion and could potentially participate in the pathogenesis of AD [33]. Examination of AD and ND age-matched control individuals demonstrated that in the former group there was significant diastolic dysfunction revealed by impaired transmitral flow efficiency of diastolic filling [34]. Intriguingly, carotid duplex ultrasound showed a significant decrease in diastolic blood flow velocities (cm/sec) in the common carotid, carotid bulb and internal carotid artery in AD patients when compared to age-matched NDC [35]. In addition, the cumulative effects of subclinical diastolic and systolic dysfunction and decreased cardiac output prior to overt cardiac failure may result in sustained brain hypoperfusion and consequent structural and functional changes conducive to dementia [32]. Cardiovascular diseases such as atherosclerosis of the cerebral arteries and diffuse brain microvascular disease in which hypertension and hypoperfusion play an important role, appear to be more prevalent in younger AD cases than in ND age-matched controls [35], [36]. Furthermore, a heavy vascular amyloid burden in the cerebral cortex of AD patients causes severe perfusion disturbances in the white matter, due to blockage of the periarterial spaces that drain the interstitial fluid, resulting in dilation of the white matter perivascular spaces (etat criblé), hydrodynamic stagnation of extracellular fluid and retention of noxious substrates [37]. Support for a secondary white matter loss in AD comes from studies that have found a negative correlation between NFT load/Braak stage and white matter cell count and blood vessel count [38].

### II. ELISA quantifications

**Figure 3:**
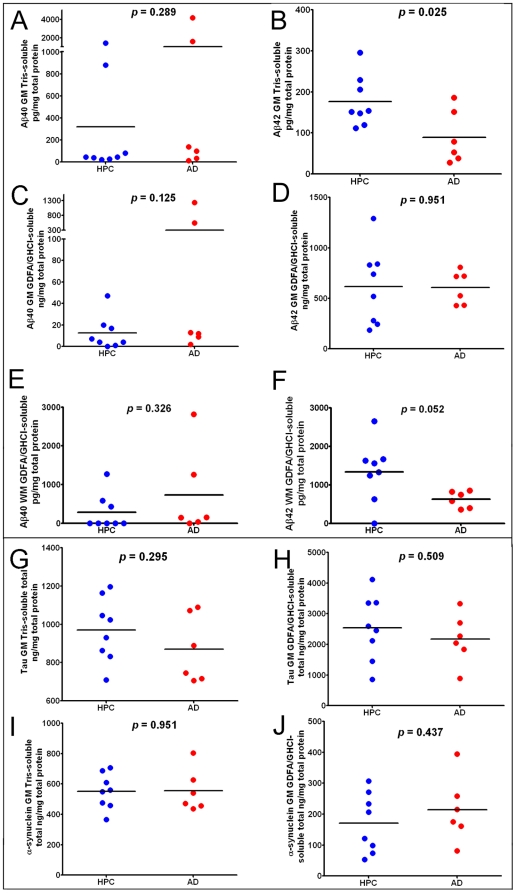
ELISA scatter plots of Aβ40, Aβ42, tau and α-synuclein levels observed in the frontal lobes of oldest-old HPC and AD cases. A-D and G-J gray matter included 8 ND-HPC and 6 AD cases. The white matter analysis in E and F also contained 8 ND-HPC and 6 AD cases. All values are adjusted for total protein. The statistical analysis used was an unpaired, 2-tailed t-test. Aβ  = amyloid-beta; GM  =  gray matter; WM  =  white matter; GDFA  = 90% glass distilled formic acid; GHCl  =  5M guanidine hydrochloride; HPC  =  non-demented high pathology controls; AD  =  Alzheimer's disease.

Immunoassays for Aβ40 and Aβ42, tau and α-synuclein were performed for all ND-HPC and AD cases, as illustrated in the plots shown in **Figure 3** **.** The enormous range of variability that exists among human subjects is reflected in the scatter plots of **Figure 3**. In conformity with the neuropathological observations, quantitation of gray matter soluble and insoluble Aβ peptides demonstrated no statistical differences with the exception of Aβ42. In the Tris-soluble gray matter samples **(** **Figure 3B** **)**, the ND-HPC demonstrated a greater mean value of Aβ42 (ND-HPC = 177 pg/mg total protein; AD = 89 pg/mg total protein; *p* = 0.025). The white matter Aβ42, solubilized with GDFA/GHCl, demonstrated significant differences (*p* = 0.052) between the ND-HPC and the AD groups **(** **Figure 3F** **)**. Intriguingly, the Aβ42 levels were significantly higher in the ND-HPC gray matter and white matter versus the AD cases (**Figure 3B and 3F**), even though they were not demented. A comparison between ND-HPC and AD white matter Aβ40 was non-significant (*p* = 0.326; **Figure 3E**), in spite of the fact that the 2 cases that had elevated Aβ40 in the gray matter (# 10 and # 14, **Table 1**) also contained exaggerated quantities of this peptide in the white matter. Immunoassay quantification of total tau and α-synuclein **(** **Figure 3G, 3H, 3I and 3J** **)** did not show any significant differences between the two groups. Setting aside the increased load of CAA, the presence of similar or higher Aβ in the ND-HPC neuropathological and biochemical Aβ brain parenchymal burdens suggest that the abundance of amyloid plaques alone is not directly responsible for the emergence of AD dementia.

**Figure 4:**
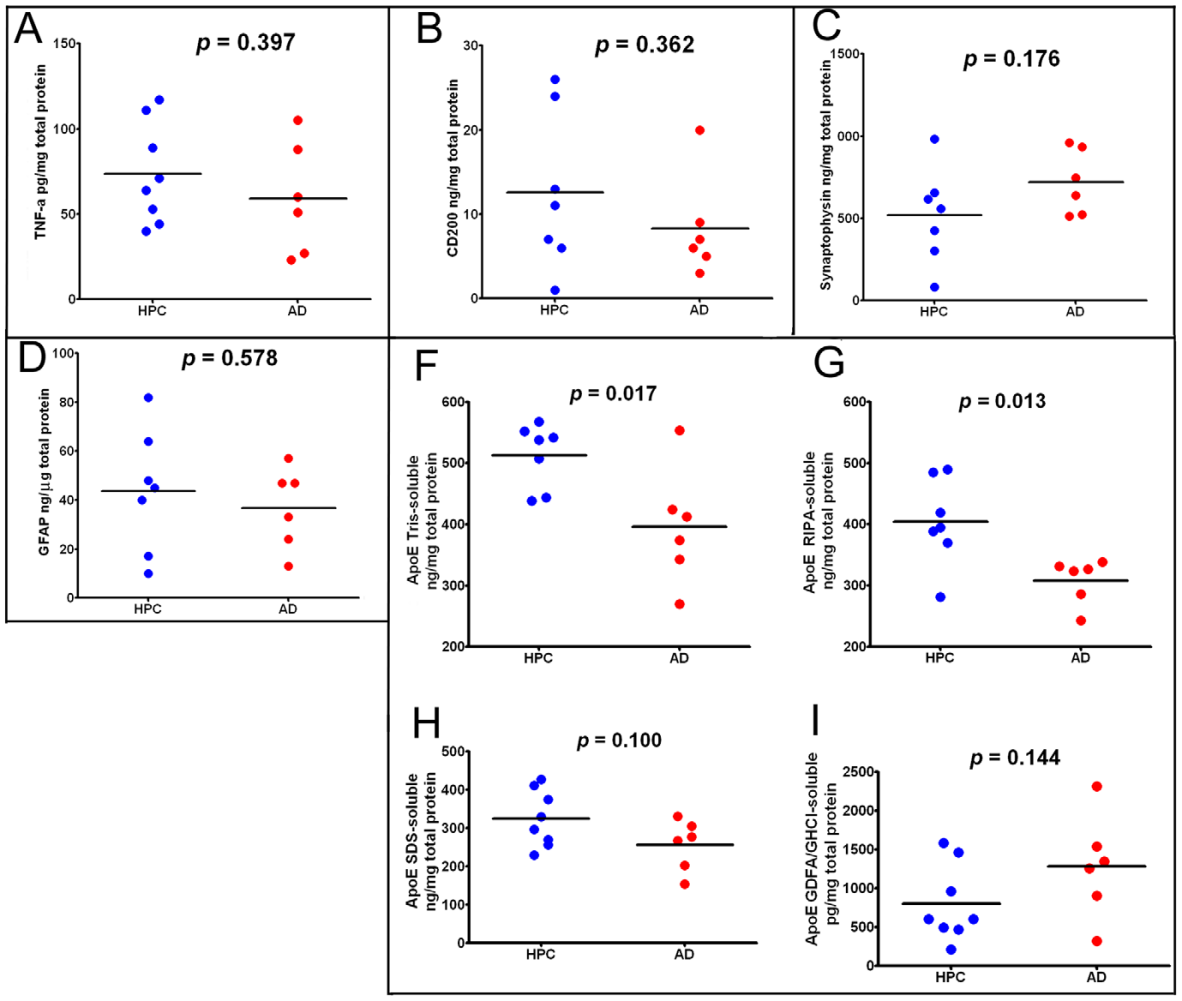
ELISA scatter plots of frontal lobe gray matter proteins. **A**) TNF-α homogenized in HEPES buffer; **B**) CD200 homogenized in RIPA buffer; **C**) Synaptophysin homogenized in RIPA buffer; **D**) GFAP homogenized in RIPA buffer; **F**) ApoE homogenized in Tris buffer; **G**) ApoE homogenized in RIPA buffer; **H**) ApoE homogenized in 5% SDS buffer; **I**) ApoE homogenized in GDFA/GHCl. All values were adjusted for total protein. The statistical analysis used was an unpaired, 2-tailed t-test. TNF-α  =  tumor necrosis factor-alpha; GFAP  =  glial fibrillary acid protein; ApoE  =  apolipoprotein E; GDFA  =  glass distilled formic acid; GHCl  =  guanidine hydrochloride; HPC  =  non-demented high pathology controls; AD  =  Alzheimer's disease.

The amounts of gray matter inflammatory TNF-α cytokine were not significantly different between the ND-HPC and AD cases **(** **Figure 4A** **)**. In addition, as shown in **Figure 4B** **,** the immunosuppressive protein CD200 was decreased in the AD compared to the ND-HPC, although the levels did not reach statistical significance (*p* = 0.362). CD200 is a highly glycosylated cell surface protein whose only known function is as a ligand for CD200 receptor. This difference may in part be responsible for better neuroprotection. CD200 is expressed primarily in neurons and oligodendrocytes [39], but has also been identified in astroglia and endothelial cells [40]. In the human brain, CD200 as well as its microglia receptor are decreased in those regions with abundant AD pathology. The synaptic vesicle marker synaptophysin was not significantly different between AD and ND-HPC when determined by ELISA (**Figure 4C**, *p* = 0.176) or Western blots (see below for discussion). Likewise, GFAP did not show any significant differences between the two cohorts under investigation **(** **Figure 4D** **)**. This intermediate filament protein is the principal structural molecule of astrocytes, the most abundant cell type in the CNS and main homeostatic modulator of neuronal function, where it regulates motility and shape and is substantially expressed as a response to trauma, chemical injury and neuroinflammation [41], [42]. GFAP has been found to be increased as the result of astrogliosis in dementia [43]. Astrogliosis is probably present in both oldest-old groups examined in the current study due to the profuse amyloid and NFT insults. GFAP has the distinction of being one of the most abundant proteins in the brain and can accumulate an enormous quantity of post-translational modifications such as phosphorylation and N-and O-glycosylations resulting in a complex array of isoforms. This intricacy, in the realm of AD and ND conditions, has been elegantly explored through proteomic analysis by Korolainen et al. [44].

Quantitative ELISA and Western blot (see below) analyses demonstrated reduced ApoE levels in AD compared to the ND-HPC. We analyzed Tris, RIPA, 5% SDS and GDFA/GHCl soluble ApoE by ELISA **(** **Figure 4F, 4G, 4H, 4I** **,** respectively). In agreement with Western blot data, ApoE was significantly reduced in Tris- and RIPA-buffer soluble fractions by ∼20% in AD compared to ND-HPC **(** **Figure 4F and 4G** **)**. The 5% SDS-soluble ApoE was reduced as well, but did not reach significance. Interestingly, ApoE tended to be higher in the GDFA/GHCl-soluble fractions in AD samples compared to ND-HPC, but also did not reach significance (see Western blot section for additional discussion of ApoE).

### III. Western Blots appraisals

**Figure 5:**
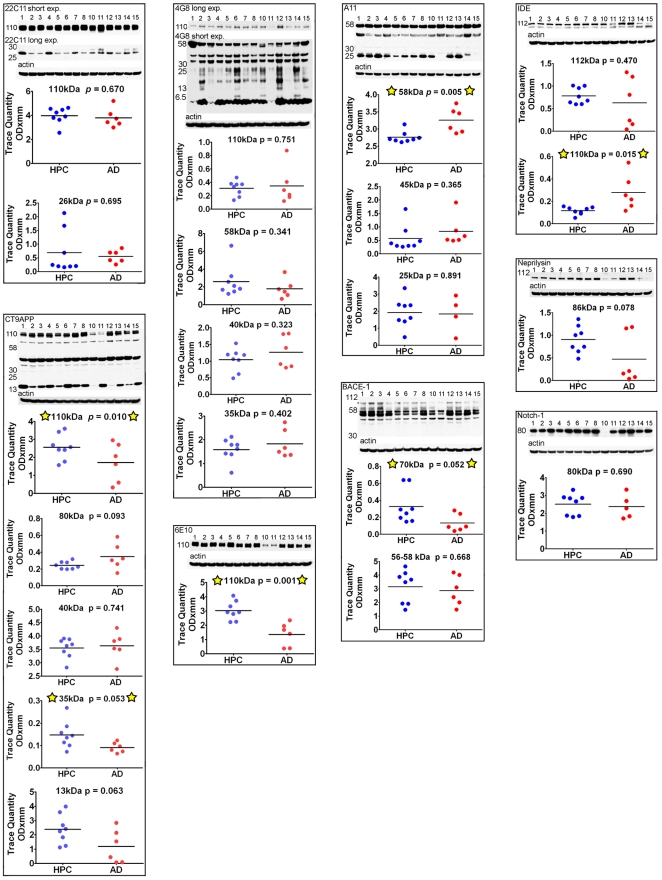
Western blots of APP/Aβ metabolism-related molecules. Five % SDS was used to homogenize the brain tissue for the CT9APP, 4G8 and 6E10 antibody blots, while the remaining blots used RIPA buffer as homogenizing medium. A total of 40 µg of total protein was loaded into each lane. Unpaired, 2-tailed t-tests were used for statistical analysis. The primary and secondary antibody descriptions and sources a given in **Table 2**. The relative abundance of the proteins were densitometrically estimated. Each blot was reprobed with actin as a loading control, as shown at the bottom of each primary antibody Western blot. The numbers to the left of the Western blots are the molecular weight in kDa. HPC  =  non-demented high pathology controls; AD  =  Alzheimer's disease; exp  = exposure.

A battery of antibodies **(** **Table 2** **)** was utilized to assess proteins that have been found to be altered in AD. For a final quality control of total protein loading, actin was used as an internal standard as shown at the bottom of each of the Western blots **(** **Figures 5** **,** **6** **, and** **7** **)**. Interestingly, the total protein values present in ∼100 mg of wet weight per ml in each of the individual specimens utilized was 14% lower in the AD group. Although this difference was not statistically significant (*p* = 0.09), it nevertheless suggests a trend in which the ND-HPC have slightly more protein per unit of brain weight than the AD specimens. This finding may also be an indication of the general loss of protein and concomitant increase in water content in the gray matter of AD subjects resulting from a chronic and emaciating neurodegenerative disorder.

**Figure 6:**
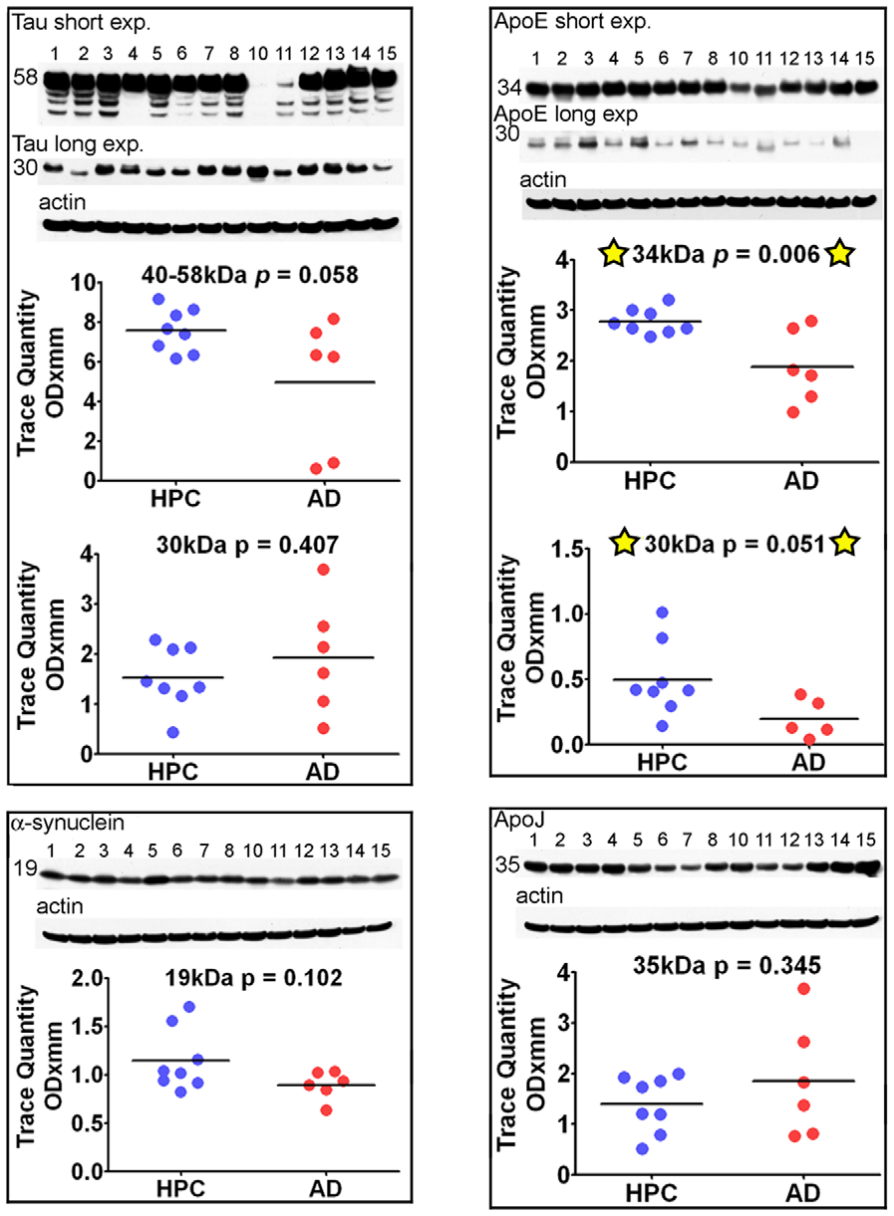
Western blots of tau, ApoE, α-synuclein and ApoJ. A total of 20 µg of total protein was loaded into each lane for the ApoE blot, while all other blots contained 40 µg of total protein. Unpaired, 2-tailed t-tests were used for statistical analysis. The primary and secondary antibody descriptions and sources are given in **Table 2**. The relative abundance of the proteins were densitometrically estimated. Each blot was reprobed with actin as a loading control, as shown at the bottom of each primary antibody Western blot. The numbers to the left of the Western blots are the molecular weight in kDa. HPC  =  non-demented high pathology controls; AD  =  Alzheimer's disease; exp  =  exposure.

**Figure 7:**
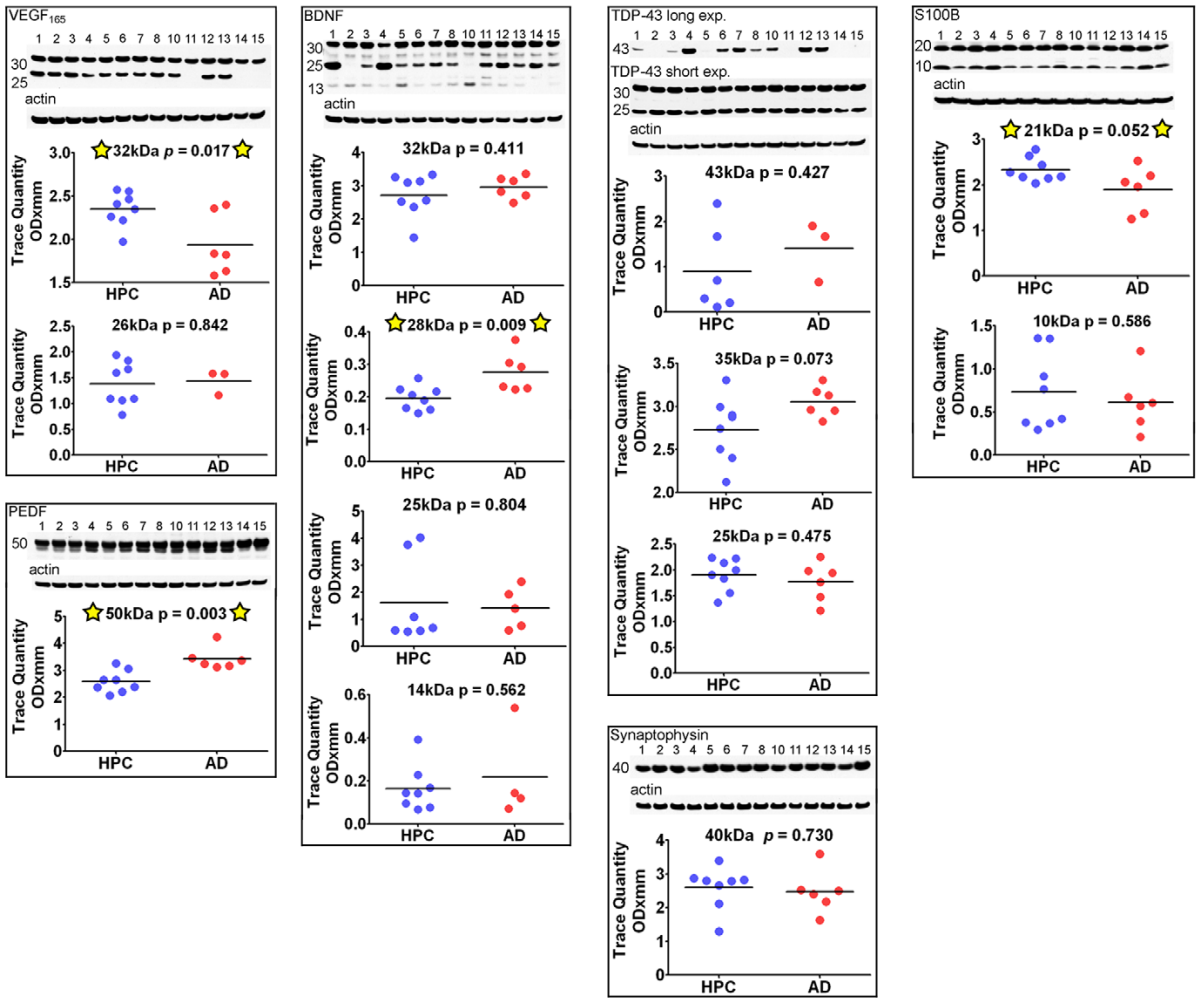
Western blots of VEGF, PEDF, BDNF, TDP-43, synaptophysin and S100B. Forty µg of total protein was loaded onto VEFG, BDNF and TDP-43 acrylamide gels. The PEDF and synpaptophysin blots contained 20 µg and 10 µg of total protein, respectively. Unpaired, 2-tailed t-tests were used for statistical analysis. The primary and secondary antibody descriptions and sources are given in **Table 2**. The relative abundance of the proteins were densitometrically estimated. Each blot was reprobed with actin as a loading control, as shown at the bottom of each primary antibody Western blot. The numbers to the left of the Western blots are the molecular weight in kDa. HPC  =  non-demented high pathology controls; AD  =  Alzheimer's disease; exp  =  exposure.

#### APP/Aβ, protease-related proteins, tau and α-synuclein

**Figure 5** illustrates the results obtained by probing with antibodies related to APP/Aβ processing and metabolism which included: 22C11, CT9APP, 4G8, 6E10, A11, β-site APP cleaving enzyme (BACE-1), insulin degrading enzyme (IDE), neprilysin and Notch-1. Significant differences between the ND-HPC and AD groups were only observed with the CT9APP antibody for the full length APP 110 kDa protein and 35 kDa peptides (*p* = 0.010 and 0.053, respectively). Likewise, the 6E10 antibody detected differences between the two cohorts in the full length APP 110 kDa protein (*p* = 0.001) while the 4G8 antibody showed no significant deviations. The results suggest that relative to the AD values, the ND-HPC APP holoprotein was elevated, as detected by the CT9APP and 6E10 antibodies, suggesting greater abundance of important APP-derived peptides such as sAPPα, sAPPβ, APP-carboxy-terminal fragment and the APP intracellular domain which have multiple neurotrophic roles, intracellular adaptor protein interactions and transcriptional functions [45]–[47]. However, differences in APP were not demonstrated with the 4G8 and 22C11 which may be explained by different antibody affinities or the fact that the 22C11 also detects APLP2 [48]. Another Aβ related molecule which also showed differences with specific antibodies between the two groups was the Aβ*56 oligomer detected by the A11 antibody that targets a variety of amyloids rich in β-sheets (*p* = 0.005). To explain the pathogenesis of AD, emphasis has been given to the presence of the Aβ*56 [49], an apparently soluble dodecameric Aβ aggregate with a ∼56 kDa M_r_ that can be detected by the A11 antibody. Although this oligomer may bolster the contention that aggregated Aβ acts as a specific neurotoxic molecule in AD, this putative soluble oligomeric Aβ has not been rigorously characterized in the human brain. In addition, the A11 antibody is not specific for Aβ since it also detects other oligomeric β-sheet conformations in a diverse number of amyloid proteins [50].

Additionally, the amounts of the β-secretase (BACE-1), a molecule extensively post-translationally modified in its mature form [51], are increased in the ND-HPC (70 kDa peptide; *p* = 0.052). Others have reported BACE-1 to be increased in AD brains (reviewed in [52]). Of the Aβ related proteolytic enzymes only the low molecular mass form of IDE 110 kDa was significantly lower (*p* = 0.015) in ND-HPC, while neprilysin and the γ-secretase target multifunctional molecule Notch-1 demonstrated no differences between the 2 cohorts of nonagenarians. The significant rise in IDE in AD versus ND-HPC could result in an elevated degradation of insulin thereby making glucose less available for energy metabolism. Alternatively, in AD, the relative enhancement of IDE may be a reflection of an inactivated enzyme by monomeric/dimeric soluble Aβ peptides [53]. Interestingly, in comparison with age-matched controls, AD brain homogenates and microvessels have a lower IDE activity [54]–[56], which may result from oxidation by reaction with 4-hydroxy-2-nonenal [57], [58]. On the other hand, these findings may simply reflect better brain structural and metabolic conditions in the nonagenarian ND-HPC.

In conformity with Aβ immunoassay observations, Western blots of tau and α-synuclein did not show significant deviations between AD cases and ND-HPC **(** **Figure 6** **)** although the 40–58 kDa tau isoforms had borderline significance (*p* = 0.058). Phosphorylated tau was detected with PHF-tau AT8 and PHF-tau AT180 antibodies and was only visible in AD # 13 and # 14 cases (data not shown). A previous study also found that AT8 immunoreactivity was limited to advanced AD stages [59].

#### Apolipoproteins

Of the two lipoproteins studied, ApoE and ApoJ **(** **Figure 6** **)**, only the former demonstrated a statistical difference between the two populations studied, being significantly diminished in the AD group: (ApoE 34 kDa and 30 kDa peptides; *p* = 0.006 and 0.051, respectively). Both Western blot and ELISA data indicate that both the native ApoE and its ∼30 kDa degradation product are decreased in AD, potentially impacting the multiple functions of this protein such as the distribution of lipids, neurite outgrowth, neurodegeneration, tau phosphorylation, oxidative activity, lysosomal function, cholesterol efflux and amyloid deposition and clearance [60]. There were no correlations between the ApoE genotype and the amounts of this molecule, although our sample size was too small to reach meaningful conclusions. The ApoEε4 genotype has been shown repeatedly to be the greatest known genetic risk factor for sporadic AD, yet the exact mechanism of its contribution to AD has not been elucidated. Cross-sectional data suggest that significantly decreased plasma ApoEε4 levels correlate with AD pathology levels assessed by PiB-PET [61]. ApoE levels have been shown to be reduced in ApoE mouse models of amyloidosis as well as in AD human subjects [25]. It has also been suggested that ApoEε4 is degraded at a higher rate than other variants [62]. We demonstrate small, but significant decreases in Tris, RIPA- and 5% SDS-soluble ApoE in AD cases, in both Western blot and immunoassay, when compared to ND-HPC. Interestingly, we found a trend towards higher ApoE in the GDFA/GHCl-soluble fractions by ELISA in AD samples compared to ND-HPC **(** **Figure 4I** **)**. Statistical significance was not reached due to the amount of variability in these fractions. Since ApoE co-localizes with Aβ in CAA [63], it is possible that decreased soluble ApoE in AD fractions is the result of its selective sequestration by vascular amyloid deposits. Similarly, the increased levels of insoluble ApoE in AD versus ND-HPC may result from the 2-fold more abundant vascular amyloid observed in our AD oldest-old cases. Moreover, recent investigations suggest that the ApoEε4 isoform is less able to clear Aβ from the brain, thus contributing to dementia [64].

#### Neurotrophic and vascular-related factors

Biochemical alterations in the AD brain's circulatory system promote changes in vascular structure, blood-brain barrier disturbances and ultimate microvessel demise [65]–[67]. These alterations prompted our investigation of vascular endothelial growth factor (VEGF) and pigment epithelium-derived factor (PEDF). As depicted in **Figure 7**, the Western blots/scanning densitometry results obtained by specific antibodies to VEGF and PEDF demonstrated significant differences between the ND-HPC and AD groups for the 32 kDa peptide (*p* = 0.017) and 50 kDa peptide (*p* = 0.003), respectively. The specific immunohistochemical reactivity of VEGF has been found to be elevated in AD where it has been localized to astrocytes, microvessels and amyloid plaques [68]–[70]. In the present investigation the pro-angiogenic VEGF was significantly decreased in AD cases relative to ND-HPC. The relative increase of this factor in the ND-HPC may reflect its neuroprotective effects when confronted with brain hypoperfusion, glucose deprivation [71]–[73] and the anti-angiogenic activity of Aβ [74]. The decreased levels of VEGF are in contrast with the concomitant increase of the anti-angiogenic PEDF in our AD specimens, a factor that naturally decreases in normal aging [75], [76]. In the AD brain, PEDF has a strong immunoreactivity in cortical neurons and astrocytes. This elevation may be explained as a defense response in AD, since PEDF has potent anti-inflammatory, anti-oxidant, anti-thrombotic, and neuroprotective properties [77]–[81]. However, an increase in PEDF may also have a negative function by preventing blood vessel formation and inducing apoptosis in proliferating endothelial cells [82]–[85], eventually leading to ischemia and neurodegeneration. VEGF and PEDF apparently have paradoxical functions on the microcirculation, since capillary permeability is increased by VEGF and inhibited by PEDF [86]. The imbalance between VEGF and PEDF has also been observed in other human diseases [87]–[91]. Interestingly, in two previous proteomic studies of CSF biomarkers, performed in our laboratory, in which neuropathologically confirmed AD and ND cases were examined by 2-D electrophoresis proteomic methodologies, PEDF was significantly increased in the CSF of an AD pool [92], [93].

Brain-derived neurotrophic factor (BDNF) is a powerful growth factor that stimulates neuronal function, prevents cell death in adulthood and is thought to be deficient in AD. Administration of BDNF into mouse models of AD, aged rats and lesion-induced primate models apparently restored learning and memory and prevented or delayed neuronal death (reviewed in [94]), suggesting BDNF as a good candidate for neurodegenerative disease clinical trials. However, investigations of BDNF levels in patients with AD and deletion studies of BDNF in mice have been contradictory (reviewed in [95]). In our Western blots, the 32 kDa N-glycosylated and glucosulfated forms of the pro-BDNF yielded no significant differences between AD cases and ND-HPC. This molecule is normally cleaved to yield the mature 14 kDa protein that was faintly visible in our oldest-old population. Of the 4 bands detected by the BDNF antibody only the 28 kDa yielded significant differences between AD and ND-HPC being increased in the former group (**Figure 7** **;** *p* = 0.009). This molecule may represent a truncated form of pro-BDNF that is aberrantly processed [96].

Multifunctional molecules. TDP-43, an important molecule with multifunctional RNA binding functions, apparently plays an important role in several neurodegenerative disorders, including AD, by generating intracellular inclusions [97], [98]. It is normally found in the nucleus, but under pathological conditions, moves to the cytoplasm where it is ubiquitinated, phosphorylated and cleaved to generate C-terminal fragments (reviewed in [99]). Interestingly, this molecule is elevated in traumatic brain injury [100]. No statistical differences between the two groups were detected for the transcriptional factor TDP-43 although half of the AD cases did not have detectable TDP-43, while this was true in only two of the 8 ND-HPC **(** **Figure 7** **)**. All groups had TDP-43 fragments [101], [102], including the C-terminal 35 kDa which was marginally elevated in AD (**Figure 7** **,** *p* = 0.073).

Similarly the presynaptic vesicular marker synaptophysin did not show statistical differences by ELISA **(** **Figure 4C** **)** which was confirmed by Western blot **(** **Figure 7** **)**. This was an unexpected observation since by immunocytochemistry there is a remarkable loss of synapses (∼45%) in AD cases when compared to ND controls [103], although our ND-HPC are uniquely different from normal ND controls in that it has an amyloid plaque burden similar to AD. Our own Western blot experiments confirmed a significant reduction of synaptophysin in a younger population of AD subjects (n = 31) when compared to ND age-matched controls (n = 22) (*p* = 0.018; A.E. Roher, unpublished observations). We did not quantify the number of synapses in our specimens, but the observation of a lack of significant difference between synaptophysin levels of ND-HPC and AD groups presents a logical conundrum. However, synaptophysin is a marker for synaptic vesicles that is extrapolated as a proxy to reveal the conditions of the synapses. This assumes physiological equivalence between the two groups, an assumption that may be confounded given the capacity for compensation under slowly emerging stressful conditions. Interestingly, synaptophysin is not essential for neurotransmitter release [104], although mice lacking synaptophysin develop behavioral and learning dysfunctions [105].

S100B is a 92 amino acid long calcium binding protein that is functionally expressed as a 21 kDa homodimer and is produced by astrocytes around blood vessels. This molecule was significantly increased in ND-HPC relative to AD (*p* = 0.05) **(** **Figure 7** **)**. S100B is an important cellular mediator of protein phosphorylation, protein degradation, cell locomotion, regulation of transcription factors, cell proliferation and differentiation, cytoskeleton assembly, regulation of enzyme activities and receptor function [106]–[108]. S100B has also been linked to survival of neurons and when generated at micromolar concentrations, enhance the production of inflammatory cytokines [107]. In addition, S100B is considered a marker of brain damage and neurodegeneration since it is elevated in global hypoxia, ischemia and hemorrhagic stroke [106], [107], [109]. It has also been associated with the density of amyloid plaques [110], [111] and it is increased in AD CSF [112], [113]. Our observation of a modest elevation of S100B in ND-HPC could be interpreted as a positive effect since this molecule has, as mentioned above, a large number of beneficial functions that may have a role in the prevention of dementia [114]. Furthermore, S100B is a potent neuroprotective factor for cholinergic neurons during oxygen/glucose deprivation [115].

### Summary and conclusions

Our goal was to evaluate the biochemical differences that distinguish the oldest-old AD population from ND-HPC. Within the framework of the multifaceted pathogenesis of AD our data suggest a compromised brain perfusion as one of the underlying causes of dementia. The brain samples showed that the severity of WMR in the AD group was over 2 times higher than the corresponding value in the ND-HPC cases. A general decline in cerebral blood flow observed in AD [116], [117] is apparently associated with loss of white matter axons and demyelination [38], [118]. When compared to a ND population, AD patients revealed a significant decrease in diastolic flow velocities (cm/sec) in the common and internal carotid arteries [35] and a decreased total and regional cerebral blood flow volumes [119]–[123]. The AD brain also exhibits increased vascular resistance [35] a manifestation of diffuse microvascular disease. A dysfunctional microcirculation in AD is supported by our observations related to decreased pro-angiogenic VEGF and increased anti-angiogenic PEDF, relative to the ND-HPC, which would restrict the ability of *de novo* vessel formation that could alleviate brain hypoperfusion. In addition, our oldest-old AD cases also had a significant decrease of S100B, an important multifunctional regulatory molecule [106]–[108]. In line with a global circulatory compromise in AD, there is an increased dilation of the white matter perivascular spaces (*etat criblé*) suggesting interstitial fluid stagnation and compromised cerebral venous outflow [37]. These data are supported by an increase in the load of vascular amyloid (total CAA score) that was on the average 2 times more abundant in AD cases than in the ND-HPC, further endorsing the contention of a compromised cerebral microvasculature, disturbed BBB and dysfunctional brain perfusion. Other studies of the oldest-old populations have also found unusual blood vessel architecture and function in AD cases compared to ND-HPC (reviewed in [5]).

Four out of the six AD cases studied showed NFT in the frontal cortex while none of the ND-HPC exhibited these lesions, reminiscent of a better preservation of the associative, executive and short-term/working memory functions in the latter group. Overall total NFT scores were about 25% higher in the AD subjects than in the ND-HPC indicative of a conserved neuronal morphology and function in the latter group. The lack of quantitative significance in the total amount of tau between the 2 groups under study emanates from the wide range of variability that characterizes tau and NFT pathology in the elderly population. Likewise, only Aβ42 revealed significant differences, but intriguingly was elevated in the ND-HPC. These data support the contention that the Aβ burden *per se*, whether soluble or insoluble, is not the decisive factor in determining the dementia status in the oldest-old subjects.

These observations are very revealing and instructive about parameters, other than or in addition to Aβ and amyloid plaque deposition, that may contribute to the conserved cognitive integrity of the ND-HPC. The lack of any clear pathological and biochemical demarcation between demented and ND groups suggests that the near exclusive focus on amyloid plaques and their components, long presumed to play dominant roles in cognitive failure, may be misguided. Along the process of aging, multi-system decay and failure to adapt and repair play a decisive role in the development of dementia. Complex environmental and molecular pleiotropic interactions are likely to govern parameters such as time of onset of disease and severity among affected individuals. These issues are well illustrated by the disease modifying effects of the ApoEε4 genotype on numerous essential functions. Characterization of pathologically deviant molecules as well as of those that promote healthy mental aging will enormously help in the identification of new targets for therapeutic interventions that will prevent, delay the onset or mitigate the clinical progression of this devastating dementia.
